# Deforestation and apparent extinctions of endemic forest beetles in Madagascar

**DOI:** 10.1098/rsbl.2007.0043

**Published:** 2007-03-06

**Authors:** Ilkka Hanski, Helena Koivulehto, Alison Cameron, Pierre Rahagalala

**Affiliations:** 1 Department of Biological and Environmental Sciences, PO Box 65, 00014 University of HelsinkiFinland; 2 Department of Environmental Science, Policy & Management, 137 Mulford Hall, University of CaliforniaBerkeley, CA 94720-3114, USA; 3 Département d'Entomologie, Faculté des SciencesBP 906, Université d'Antananarivo, Madagascar

**Keywords:** Madagascar, forest loss, tropical extinctions, dung beetle, *Helictopleurini*

## Abstract

Madagascar has lost about half of its forest cover since 1953 with much regional variation, for instance most of the coastal lowland forests have been cleared. We sampled the endemic forest-dwelling Helictopleurini dung beetles across Madagascar during 2002–2006. Our samples include 29 of the 51 previously known species for which locality information is available. The most significant factor explaining apparent extinctions (species not collected by us) is forest loss within the historical range of the focal species, suggesting that deforestation has already caused the extinction, or effective extinction, of a large number of insect species with small geographical ranges, typical for many endemic taxa in Madagascar. Currently, roughly 10% of the original forest cover remains. Species–area considerations suggest that this will allow roughly half of the species to persist. Our results are consistent with this prediction.

## 1. Introduction

Nearly two-thirds of all described species of animals and plants are insects, of which roughly 45% are beetles, but the level of threat imposed by the global environmental changes to beetle and insect diversities remains poorly documented. Of the described species of insects and beetles, only 0.07 and 0.02% have been classified as globally extinct or threatened, respectively, compared with 24% for mammals and 12% for birds (IUCN 2004; Birdlife 2006). Almost certainly, the figures for insects reflect lack of knowledge rather than lack of threat to insects. In the parts of the world that are best studied, insects are equally as threatened as vertebrates (Thomas *et al*. 2004) or even more threatened (Stein & Flack 1997). In Finland, with a beetle fauna of 3640 species, 12.1% of the species are classified as nationally extinct (1.6%) or threatened (10.5%), compared with 15.3% in birds, 17.0% in mammals and 15.4% in vascular plants (Rassi *et al*. 2001).

Knowledge of tropical insects is particularly limited. One exception is the fauna of Singapore, which has been studied since 1819 (Brook *et al*. 2003). The historically documented extinction rate is 34% for birds, 43% for mammals, 26% for vascular plants and 38% for butterflies (Brook *et al*. 2003). Again, there is no difference between vertebrates, plants and butterflies.

Here, we examine apparent extinctions in a well-studied group of *ca* 60 endemic forest-inhabiting dung beetles in Madagascar. Our extensive sampling across Madagascar during 2002–2006 yielded 54% of the described species, as well as four new ones. We analyse the factors that explain whether a previously known species was included in our samples or not. We refer to the latter species as ‘apparently extinct’ for short. We are particularly interested in examining whether regional forest loss may explain apparent extinctions. The annual rate of deforestation has been 1.4% between 1953 and 1993 and 2.0% between 1993 and 1999 (Dufils 2003), and hence roughly half of the forest cover has been lost in the past 50 years. The endemic forest dung beetles have small ranges (Viljanen *et al*. in preparation), as have many other taxa in Madagascar (Wilmé *et al*. 2006), and hence it is possible that many species have become (effectively) extinct due to a regionally high rate of forest loss.

## 2. Material and methods

**Figure 1:**
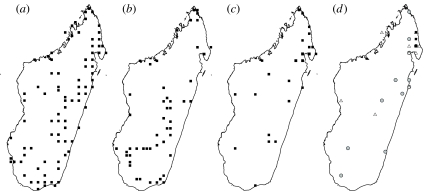
Maps showing (*a*) the historical sampling localities during 1875–1990, (*b*) our sampling localities during 2002–2006, (*c*) the sampling localities of 21 apparently extinct species apart from *H. undatus* and (*d*) the localities for *H. undatus* prior to 1900 (grey circles), during 1901–1950 (open triangles) and during 1951–1973 (black squares). Localities are mostly shown with resolution of 0.5°.

The taxonomy of the endemic tribe Helictopleurini (Coprinae, Scarabaeidae) is well known (Lebis 1960; Paulian 1986 and other papers; Montreuil 2005, in press and other papers). We recorded the sampling information for all specimens in the main collections of the Paris National Museum of Natural History. The collections include 51 species for which locality information is available, sampled from 126 distinct localities (figure 1*a*) during 1875–1990 (3341 specimens). Half of the specimens have been collected prior to 1926 but almost none since the mid-1970s. The remaining nine species either lack sampling information (three species) or there are no specimens in Paris. We suspect that several of these latter species should be synonymized with the better known species.

During the years 2002–2006, we sampled Helictopleurini using dung and carrion-baited traps. Our sample of 4880 specimens was collected from 61 localities (figure 1*b*), including larger samples from Ranomafana National Park (NP), Masoala NP, Makira Reserve, Andasibe NP, Ambila-Lemaintso, Manombo reserve, Isalo NP, Zombitse-Vohibasia NP and Andahohelo NP. Smaller samples were collected by the personnel of forest reserves in 52 localities across Madagascar using trapping kits provided by us. Our samples include four new species (Montreuil 2005, in press).

A single map of forest cover change between the years 1970, 1990 and 2000 was provided by Conservation International (CI) at approximately 30 m resolution (Harper *et al*. 2005, unpublished data). This map was reclassified to single out forest cover for the year 2000. The main sources of data were the Inventaire Ecologique et Forestaire National (IEFN) classification of Landsat Thematic Mapper 5 data for the year 1993 and estimates of forest cover for the year 1999 carried out by the Joint Research Centre (JRC)–Space Application Institute, Ispra, Italy, using SPOT-4 data. Where possible, CI used additional SPOT images to add details in the regions covered by dense cloud in the original IEFN and JRC images.

CI also provided a digitized version of the 1953 forest cover map produced by Humbert *et al*. (1965), rasterized at the same resolution as the 2000 map. The original map was produced using aerial photographs and ground truthing. The 1953 study appears to have focused on mapping major forest blocks, as the map does not contain small fragments in remote areas that were present in the satellite images. We assume that the additional small fragments present in 1970 had not grown in the intervening years, and hence any forest cover present in 1970 but absent in the 1953 map was added to the latter.

The maps for 1953 and 2000 were summarized as percentage of forest cover in grid cells of 0.1° resolution (11.2 km at the equator). Using these maps, we calculated the extent of forest cover within the range of species *x* at time *t* (1953 or 2000) as

(2.1) $$F_{x,t}=(1/n)\sum \sum {\text{e}}^{-\alpha d_{ij}}A_{j},$$

where *A*_*j*_ is the percentage of forest cover in cell *j*, *d*_*ij*_ is the distance between the sampling locality *i* and cell *j* in degrees, and the second summation is over the *n* sampling localities for species *x*. Thus, *F*_*x*,*t*_ measures the average amount of forest in the surroundings of the historical sampling localities for species *x*, giving decreasing weight to cells with increasing distance from the sampling localities. We assumed *α*=10, which gives substantial (greater than 0.05) weight to distances up to 33.6 km. The absolute and relative forest losses were calculated as *F*_*x*,1953_−*F*_*x*,2000_ and *F*_*x*,2000_/*F*_*x*,1953_, respectively.

As our sampling localities do not evenly cover all of Madagascar, it is possible that we have failed to sample a species because only a few or even none of our sampling localities were within its range. To account for this, we calculated the average distance of the *n* historical sampling localities for species *x* to our sampling localities as

(2.2) $$D_{x}=(1/n)\sum \sum {\text{e}}^{-\alpha d_{ij}},$$

where *d*_*ij*_ is the distance (in degrees) from the *i*th historical sampling locality to the *j*th locality in our sampling. Since we used the value *α*=1 in this calculation, 1° (112 km) distance has the weight 0.37.

Other explanatory variables include body size, the last year when the species was sampled prior to our sampling, the (log) number of historical sampling localities, the (log) number of individuals in the Paris collections and the range of the species, defined as the distance between the two most distant historical sampling localities. As the latter three variables are all strongly correlated, we calculated the first principal component as a general measure of past commonness (PC1). PC1 accounted for 90% of variation in the three original variables.

## 3. Results

Out of the 51 species sampled prior to our work and for which locality information is available, we have sampled 29 species but failed to collect 22 other species. We ran stepwise logistic models with the explanatory variables described in §2 to explain apparent extinctions. Relative forest loss (*F*_*x*,2000_/*F*_*x*,1953_) entered the model first (table 1). The other two variables that were selected were distance to our sampling localities (*D*_*x*_) and PC1. An equally good model was obtained if PC1 was dropped from the candidate variables, in which case the last year when the species had been sampled was selected at 5% level.

Since the uncollected species cannot be included in our molecular phylogeny (Koivulehto *et al*. in preparation), we cannot critically assess possible phylogenetic bias in apparent extinctions. However, we may use the eight taxonomic species groups of Lebis (1960) as a proxy, as these groups match the clades in the molecular phylogeny reasonably well. There is no difference in the fraction of apparently extinct species among the morphological groups (*p*=0.46).

Most of the 22 species that we have not collected have been previously collected from only one (*n*=9) or two (*n*=6) localities, widely scattered across Madagascar (figure 1*c*). The most striking exception is *Helictopleurus undatus*, which has been collected from 27 localities across much of Madagascar. A closer examination shows that since 1950 this species has been restricted to a small region in the northeast (figure 1*d*), giving the impression that it gradually disappeared from its former range during the twentieth century.

## 4. Discussion

Helictopleurini are mostly relatively large, many species have colour-patterned elytra and are diurnal and easy to sample with dung and carrion-baited traps. For these reasons, Helictopleurini have been relatively well collected in the past. In our specialized sampling, only four new species were discovered, all of which appear to be very localized and rare. Four species compose only 7% of the described species, which is a small percentage for tropical insects in as large and diverse an area as Madagascar. We conclude that Helictopleurini have been sufficiently well collected in the past to warrant this analysis.

**Figure 2:**
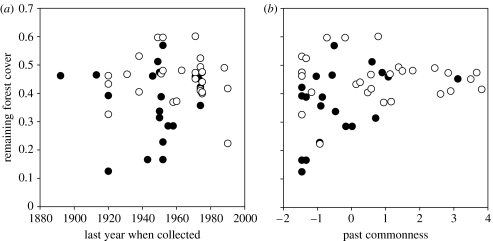
Plots showing whether a species has been collected by us (open symbols) or not (closed symbols, ‘apparently extinct’ species), depending on relative forest loss within the range of the species plotted (*a*) against the last year when the species was collected prior to our sampling and (*b*) against the past commonness of the species (PC1).

The best predictor of whether a species was collected by us or not was relative forest loss within its past range. The estimated remaining forest cover from 1953 to 2000 ranges from 10 to 60% for different species (figure 2). Species for which less than one-third of the 1953 forest cover remains tended to be apparently extinct. The other factors that had a significant effect on species' occurrence during 2002–2006 were PC1, the last year when the species had been collected prior to our sampling and the distances from the past sampling localities to our sampling sites. None of these effects is unexpected. Most of the apparently extinct species have not been collected for 50 years or longer (figure 2).

It is perhaps surprising that the past or current extent of forest within the range of the species had no significant effect on whether we collected the species or not. Admittedly, the forest maps depict a crude picture of forest cover and the past sampling localities provide inaccurate estimates of species' ranges. In this situation, long-term change rather than one-time state of the environment may better reflect species' responses. Furthermore, in many regions forest loss from 1953 to 2000 was a continuation of a longer process. Some species may have been on decline already in 1953 due to deforestation, and the occurrence of species is tracking forest loss and fragmentation with a time lag (Hanski & Ovaskainen 2002).

Forest loss and fragmentation is associated with an increasing pressure on lemurs, the most important dung producers in Madagascar. According to the fossil record, 16 large-bodied lemurs have gone extinct since human colonization *ca* 2300 years ago (Burney *et al*. 2004). The long-term and dramatic decline of *H. undatus* (figure 1*d*) may be due to high degree of resource specialization. Unfortunately, nothing is known of its biology and by now this once exceptionally widespread species may already be extinct.

Madagascar has large numbers of species with narrow geographical ranges (Wilmé *et al*. 2006). In this situation, and taking into account that Madagascar has already lost most of its forests, very large numbers of insects and other poorly known taxa may already be extinct, effectively extinct or rapidly heading towards extinction due to past and current deforestation. The current plans to expand the protected area network to six million hectares will amount to *ca* 10% of the original forest cover. Species–area considerations suggest that this will protect roughly half of the species (MacArthur & Wilson 1967). Our results are consistent with this prediction.

## Figures and Tables

**Table 1:** Stepwise logistic regression model explaining whether we have sampled a species of *Helictopleurini* (*n*=29) or not (*n*=22). (*p*-value for the full model=0.38; d.f.=47.)

explanatory variable	deviance	difference	*p*
constant	69.74		
relative forest loss	61.52	8.21	0.004
distance to our traps	53.24	8.28	0.004
past commonness	49.24	4.00	0.046
